# High-Dose Rate Salvage Interstitial Brachytherapy: A Case-Based Guide to the Treatment of Therapeutically Challenging Recurrent Vulvar Cancer

**DOI:** 10.3389/fonc.2017.00224

**Published:** 2017-09-20

**Authors:** Kelly Eileen Hughes, Christopher M. McLaughlin, Emma C. Fields

**Affiliations:** ^1^School of Medicine, Virginia Commonwealth University, Richmond, VA, United States; ^2^Radiation Oncology, Massey Cancer Center, Virginia Commonwealth University, Richmond, VA, United States

**Keywords:** vulvar cancer, brachytherapy, recurrent vulvar cancer, salvage interstitial brachytherapy, high-dose rate brachytherapy

## Abstract

Vulvar cancer is a rare gynecological malignancy with incidence rates steadily increasing over the past 10 years. Despite aggressive treatment, recurrent disease is common. Vulvar cancer recurrence poses a significant therapeutic challenge as most patients have been previously treated with surgery and/or radiation limiting the options for additional treatment. There are no consensus guidelines for the treatment of recurrent disease. Current literature supports the use of salvage interstitial brachytherapy. However, the total sample size is small. The goal of this case report is to review the current literature and to provide a guide for the use of salvage interstitial brachytherapy for recurrent disease by describing, in detail, the techniques used to treat two patients with unique cases of vulvar cancer recurrences in women with advanced disease and multiple medical comorbidities.

## Background

Vulvar cancer is a relatively rare gynecological cancer accounting for approximately 4% of gynecological malignancies (1, 2). Current incidence rates are approximately 2–3 per 100,000 people; however, these rates have been steadily increasing over the past 10 years, approximately 0.6% per year (2). However, standard treatment paradigms have been established based on older data, with little advances over time (3, 4). Surgery is the mainstay of treatment, and external beam radiotherapy (EBRT), brachytherapy, and chemotherapy can be incorporated as adjuvant, neoadjuvant, or monotherapy in rare instances, based on the stage and progression of disease (1). The prognosis of vulvar cancer is strongly dependent on lymph node involvement with 5-year survival rates varying from 91% in those with node negative disease to 52% in those with nodal positivity (1, 2).

Given these percentages, it is clear that despite aggressive treatment at diagnosis, disease recurrence is a relatively common event (1, 2). In fact, approximately 30% of patients will develop recurrent disease after primary treatment, and this number may be as high as 40–50% in patients who initially present with locally advanced disease. Most patients experience local recurrence at or near the original disease site, and few experience recurrence in the groin or distant recurrence outside of the pelvis (1, 5–9). Most recurrences will occur within two years after primary treatment; however recurrences have been documented up to 10 years later. Salvage treatment for recurrent disease is particularly difficult because most, if not all, patients have been previously treated with surgery with or without adjuvant radiation. Surgery changes the vascular and lymphatic anatomy making additional surgical or radiotherapeutic attempts less successful, and reirradiation is limited due to the proximity of dose-limiting organs such as the urethra, bladder, vagina, anus, and rectum.

Given the rarity of primary vulvar cancer, it is difficult to perform randomized prospective studies regarding treatment of recurrent disease. Because of this, there are no consensus guidelines, and no clear standard of care exists often making treatment decisions difficult and prognosis poor (1, 6, 7, 9). Interstitial brachytherapy is an elegant technique delivering high-dose, focal radiotherapy which allows for a maximal radiation dose to be delivered to the tumor with minimal damage to nearby organs at risk. High-dose rate (HDR) interstitial brachytherapy has been used as a salvage treatment, however, only 11 patients have been reported on in the literature (10, 11). One study reported on 8 salvage patients with median time to failure of 31 months and progression free survival at 1 and 3 years 100 and 62.5%, respectively. The purpose of this case report is to describe in detail the techniques used in two unique cases of vulvar recurrences in women with multiple other medical issues who were treated with salvage interstitial brachytherapy and to add to the current literature on the topic of recurrent vulvar carcinoma. Both patients discussed in this report provided written informed consent regarding participation in their respective treatments and the publication of this manuscript.

## Patient Introduction

### Patient A

**Figure 1:**
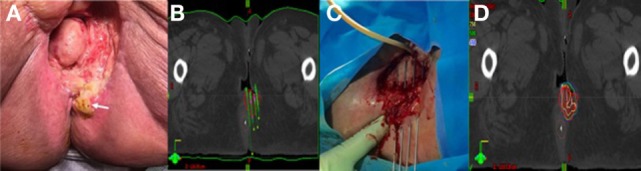
**(A)** Pretreatment image of Patient A’s posterior lesion adjacent to the anus (white arrow). **(B)** Preplanning computed tomography (CT) scan for patient A with mock-up of needle placement. **(C)** Treatment setup for patient A showing placement of guide needles (prior to flexible breast catheter placement). **(D)** Brachytherapy treatment plan for patient A (red = gross tumor, brown = rectum).

Patient A is a 65-year-old female with severe cardiovascular disease (atrial flutter requiring a pacemaker and rheumatic valve disease requiring mitral valve replacement) who was initially diagnosed in June 2015 with FIGO stage IB, grade 2 squamous cell carcinoma (SCC) of the vulva. She underwent primary surgery with a wide local excision at an outside hospital which revealed multifocal grade 2 disease with a 3.7 cm lesion on the perineal body and a 2 cm clitoral lesion with 6 and 7 mm depth of invasion and 1 and 2 mm margins, respectively. There was no lymph vascular space invasion identified and no inguinal lymph node evaluation, but both lesions had perineural invasion. Given the risk factors for primary recurrence, she underwent adjuvant EBRT receiving a total of 54 Gy in 30 fractions to include the primary, bilateral inguinal regions and the low pelvis. Within 3 months, she developed a new lesion, and biopsy revealed vulvar intraepithelial neoplasm of the right vulvar region near the midline and invasive SCC of the left vulva. The patient underwent radical vulvectomy and partial vaginectomy with pathology showing a 2.7 cm left labial grade 3 SCC with a depth of invasion of 11 mm, focally positive margins at 3 o’clock and re-resected to negative at ink. There was no lymph vascular space invasion, and no lymph nodes were resected. Not surprisingly, only 4 months after this surgery, she developed a perianal recurrence in the posterior aspect of the previous surgical site (Figure 1A). Biopsy again revealed SCC. Her only surgical option at this time was posterior exenteration and bowel diversion. In order to minimize dose to surrounding structures and prevent the need for extensive surgery, the patient was treated with HDR interstitial brachytherapy with Ir-192.

### Implant

Prior to catheter placement, the patient had a computed tomography (CT) scan to create a preplan for the implant. During CT, we placed wired markers around the tumor recurrence to delineate the gross tumor volume (GTV) and marked the anus with a BB. A preplan was created with the dose prescribed to the periphery of the GTV (contoured generously around the wire marker) (Figure 1B).

The procedure was performed in our brachytherapy suite under general anesthesia with the radiation oncologist and gynecologic oncologist. Four needle-guided flexible catheters were placed in a single plane through the 3 cm × 3 cm lesion (Figure 1C). Given the small size of the recurrence (12.7 and 2.9 cm) and the location on the perineum adjacent to the anus, flexible “plastic tube” catheters, which are usually employed for breast implants, were used for the interstitial implant.

### Treatment and Follow-up

Computed tomography simulation was performed immediately following the implant and the GTV was again generously contoured based on the preplan images and needle locations. The anus and rectum were contoured as the primary organs at risk. The patient received an interstitial dose of 25 Gy in 5 fractions over a 3-day period (Figure 1D). The dose to the anorectum was just over 2 Gy per fraction with this plan. The first treatment was given on the day of the procedure and the subsequent fractions were given twice a day on days 2 and 3 with at least 6 h between fractions. Each morning, a CT scan was taken to confirm needle placement and positioning of organs at risk. The patient was admitted to the hospital for the duration of treatment with an epidural to control pain. She tolerated the procedure well without any significant complications.

In the weeks immediately following the procedure, this patient had some perineal discomfort and mild bleeding. She did not experience any other treatment-related toxicities and did not develop any serious complications.

Unfortunately, 4 months later the patient had a biopsy confirmed recurrence at a site on the perineum outside of our treatment field. She received one cycle of chemotherapy with cisplatin and mitomycin-C before passing away from her disease. Survival was 21 months from the initial diagnosis and 6 months from the time of salvage therapy.

### Patient B

**Figure 2:**
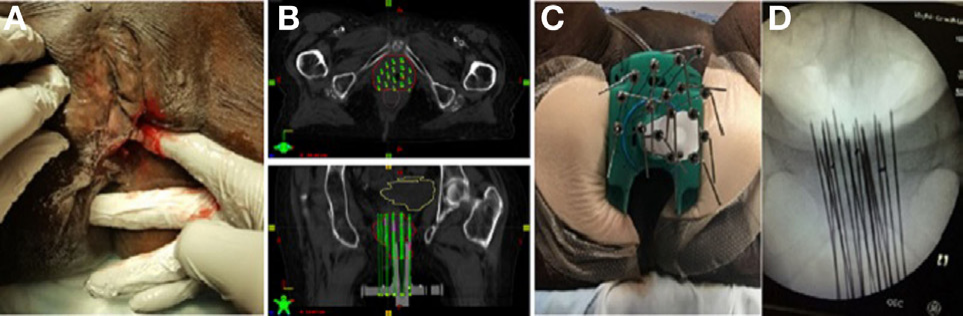
**(A)** Pretreatment image of tumor in right vulva. **(B)** Preplanning computed tomography (CT) scan for patient B with mock-up of needle placement. **(C)** Treatment setup for patient B with interstitial needles and modified Syed template to allow transrectal ultrasound. **(D)** Interstitial needle insertion under fluoroscopic guidance.

Patient B is a 90-year-old female with multiple medical comorbidities including coronary artery disease, diabetes, hypertension and a left lower extremity amputation, who was initially diagnosed with stage IVA, T3N3 SCC of the vulva. The 2 cm lesion originated in the medial aspect of the right labia and extended superiorly into the proximal vagina with fixed lymph nodes in the bilateral inguinal region. She was not felt to be a good candidate for chemotherapy or surgery secondary to her comorbidities and underwent EBRT monotherapy to a total of 60 Gy in 30 fractions. Following treatment, she represented with progressive perineal pain and was found to have a large tumor recurrence/persistence on the right vulva with complete obliteration of the vaginal canal (Figure 2A). Given her previous radiation treatment, our only option at this time was interstitial brachytherapy. She underwent treatment with HDR interstitial brachytherapy with Ir-192.

### Implant

Similarly to patient A, this patient had a preplanning CT scan for creation of a guide for the implant. The CT was done with a vaginal obturator and perineal template (Syed) in place to simulate the positioning during brachytherapy. Gross tumor was marked with radio-opaque wires and an anal BB was placed. Again, GTV was contoured generously and nearby organs were contoured with a goal of prescribing 20 Gy to the periphery of the tumor with as low a dose as possible to 2 cm^3^ of the bladder and rectum. The preplan created from the scan used 17 needles (Figure 2B).

The implant procedure was performed in the brachytherapy suite under general anesthesia. We used a modified Syed template with a custom cutout to allow for the transrectal probe for ultrasound image-guidance (Figure 2C). 17 stainless steel needles were inserted to a predetermined depth based on dosimetric planning using both ultrasound and real time fluoroscopy (Figure 2D).

### Treatment and Follow-up

**Figure 3:**
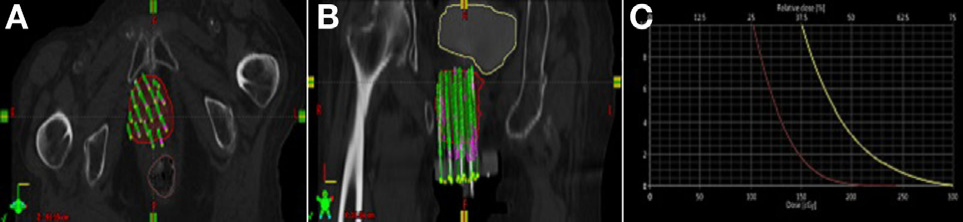
**(A,B)** Brachytherapy treatment plan for patient B (red = gross tumor, yellow = bladder, brown = rectum). **(C)** Dose volume histogram for patient B.

The treatment plan and dose volume histogram from postimplatn CT images are displayed in Figures 3A–C. Given her prior EBRT and large tumor recurrence (88 and 5.5 cm), she received an interstitial dose of 20 Gy in 5 fractions delivered over 3 days in the same fashion as patient A. The dose constraints for both patients were designed to minimized dose to the rectum and bladder. Given the 60 Gy external beam dose, the goal was to give less than 2.5 Gy per fraction to 2 cm^3^ of the rectum and less than 3 Gy per fraction to 2 cm^3^ of the bladder to keep the doses less than 75 and 85 Gy, respectively, using an equivalent dose in 2 Gy fractions.

She experienced minimal acute toxicity including pain, a urinary tract infection, and vaginal dryness which resolved by her 4-week follow-up visit. She did not experience any significant long-term complications.

In fact, she had an excellent response to treatment with near complete resolution of her large tumor. However, following treatment, she developed gradually worsening oral intake, and given her advanced age and comorbid conditions, her family had increased trouble caring for the patient. She entered home hospice 3 months after treatment.

## Discussion

This series adds to the small body of literature describing the successful treatment with this highly conformal technique. Here, we describe in detail how two unique cases were treated so that it may be replicated for other women who may also benefit.

As noted previously, there are no consensus guidelines nor is there a standard of care for treating cases of recurrent vulvar carcinoma due to the relative rarity of disease and lack of prospective studies available. Options for vulvar-confined local recurrence include wide local excision with or without radiation, radical vulvectomy, or pelvic exenteration in select cases of central recurrence (1, 2, 9). More extensive recurrences with multiple nodes and/or distant metastases generally require a combination of radiation and cytotoxic chemotherapy with or without surgery, but prior treatments, particularly prior wide field or high-dose radiation add complexity. Several current clinical trials are investigating the use of various chemotherapeutic agents in the treatment of vulvar recurrence (2, 9). While various levels of success have been reported with these options, recurrent cancers continue to have a very poor prognosis.

Surgery with wide local excision has been the most successful and most widely accepted treatment approach for recurrent disease with cure rates as high as 70% (8, 9). However, some patients are unable or unwilling to undergo surgery. The blood supply and lymphatic drainage of remaining tissue is compromised following surgery making additional surgical or radiotherapeutic attempts more complex and less successful (10, 12, 13). Patients with central recurrences require more extensive surgery which is associated with high mortality and complication rates as high as 50–75% (9, 14, 15).

Also, surgical procedures can make radiotherapy attempts more challenging. Patients who previously underwent EBRT are limited with reirradiation due to the proximity of dose-limiting organs such as the bladder or rectum which have likely already accumulated their maximal dose of radiation (10, 12). Brachytherapy may play a useful role in these cases where patients require extensive surgery or in cases where surgery is contraindicated or refused.

The use of interstitial brachytherapy in the treatment of vulvar cancer is not a new concept, but patient numbers are small with just 55 cases published in the literature (10–12, 16–20) (Table 1). However, it is clear that the approach has survived the test of time due to low rates of toxicity and reasonably high rates of efficacy.

**Table 1:** Literature review of interstitial salvage brachytherapy for vulvar cancer.

Study	Time period	Total pts	Salvage pts	Technique	Dose (Gy)	Outcome
Prempree and Amornmarn Florida (19)	1958–1977	21	21	LDR	55–85	5-year DFS 38%
Hoffman et al. Florida (18)	1985–1988	10	Unknown	LDR	70–90	3-year OS 80% all comers
Pohar et al. France (17)	1975–1993	34	15	LDR	60 (53–88)	5-year LC 19% for recurrence (vs. 80% for first presentation *p* = 0.04) 5-year OS 29% in all patients and 33% in salvage patients (*p* = 0.64)
Tewari et al. California (12)	1985–1992	11	5	LDR	28.66 (23.6–35)	MS 33 months all patients LC 2 years 82%
Dyk et al. Wash U (16)	2006–2012	50	Unknown^a^	HDR	51.3 (30–60)	1-year LC 72% all comers
Kellas-Sleczka et al. Poland (10)	2004–2014	14	8	HDR	15–43	1- and 3-year PFS for recurrent group 100 and 62.5% MS 28 months
Castelnau-Marchand et al. France (20)	2000–2015	26	3	LDR and PDR	60 (55–60)	3-year OS 81% and DFS 57% for all comers
Mahantshetty et al. India (11)	2001–2016	38	3	HDR	38.4 (35.5–46.7)	5-year OS 82% DFS 51% LC 77% for all comers

*^a^16 salvage and total, 13 vulvar; unclear how many recurrent vulvar cancers*.

*DFS, disease-free survival; OS, overall survival; LC, local control; MS, median survival*.

In these experiences, the most common complications experienced by patients included skin ulceration, vulvar edema, mucositis, and GI/GU toxicity. Of all the studies reviewed, only three salvage patients experienced severe treatment-related complications including one who developed a rectovaginal fistula and two who developed severe soft tissue necrosis (10, 12). Mahantshetty et al. also reported late toxicities in 22% including conservatively managed soft tissue necrosis, radiation proctitis, urethral stenosis, and skin and subcutaneous fibrosis (11). However, this was generalized for all patients and does not specify toxicity rates for salvage treatment. Less severe acute toxicity rates were low in all studies. Patients in studies by Pohar et al. and Prempree and Amornmarn did not experience any serious complications and reported acute toxicity rates of 14 and 0% respectively (17, 19). Both of the patients discussed in our current study avoided serious complications and experienced only mild acute toxicity that resolved with conservative treatment.

Multiple studies report favorable outcomes when using interstitial brachytherapy to achieve local control and disease free periods in these women with recurrent cancers. Two recent studies show very promising results with survival rates approaching those seen in some surgical studies. Kellas-Sleczka treated eight patients with recurrent vulvar cancer all of whom had previously undergone radical surgery, three of whom had lymph node involvement at the time of treatment. Overall survival rates and progression-free survival rates at 3 years were 80 and 62.5% respectively. However, 50% of patients relapsed with median time to failure of 31 months (10). Mahantshetty et al. treated 38 patients, 3 of whom were treated for recurrent disease, and found 5-year overall survival, disease-free survival, and local control rates of 82, 51, and 77% respectively (11). Unfortunately, the specific results for salvage patients were not reported. Patients in both of these studies were treated using HDR brachytherapy which has recently begun replacing low-dose rate (LDR) techniques which was the technique used for our patients. Compared to LDR, HDR brachytherapy is more convenient and allows for shorter treatment times and better optimization for patients with better radiation protection of the staff (11).

Older reports by Pohar, Prempree, and Tewari all used LDR brachytherapy implants with or without combined EBRT. Tewari et al. recorded disease free survival ranging from 3.5 to 4 years in four out of five patients treated for vulvar recurrence (12). Pohar et al. compared their results using brachytherapy with surgical series (17). They determined that brachytherapy results were inferior leading them to conclude that brachytherapy is a viable option only when surgery is contraindicated or refused. However, it is important to note that this is a difficult comparison to make as the patients in Pohar’s study were older, and many had medical comorbidities precluding them from surgery which may have contributed to their decreased survival rates. Another surgical study reported a 29% 5-year survival in 48 patients treated with surgery for recurrent disease, while Pohar et al. reported a 33% 5-year survival which suggests that brachytherapy may be equivalent to surgical treatment in certain recurrent cases (21). However, it is difficult to compare with such a small sample and without controlling for demographic information and tumor specifications such as size and nodal status.

Even though both of our patients ultimately succumbed to their vulvar cancer, interstitial brachytherapy was an effective and safe treatment which provided disease control and a reasonable quality of life. Our first patient was initially treated with surgery and EBRT making recurrent treatment options limited. Interstitial brachytherapy provided an alternative to exenteration by allowing us to provide high doses of radiation to the tumor bed while avoiding additional radiation damage to the bladder and rectum and avoiding the need for extensive surgery with colostomy. Boronow et al. reported good results using a combination of radiotherapy (combined external beam and intracavitary brachytherapy) and surgery as an alternative to pelvic exenteration. They reported 65.6% 5-year survival for recurrent cases with satisfactory mortality and morbidity. Additionally, they reported a 95% viscera preservation rate leading to significant improvements in quality of life (22, 23). Our experience supports this technique as an alternative to exenteration as our patient maintained good local control of disease at 6-month follow-up and did not require colostomy.

Our second patient had very few options, to begin with, given her age and multiple other comorbidities. When her disease recurred, interstitial brachytherapy was truly the only option that we had to obtain any control of her disease. Her extensive disease and obliteration of perineum and vaginal canal made the treatment approach fairly difficult and required a decent amount of creativity. The modified Syed template and transrectal ultrasound guided needle placement were unique to this patient.

## Concluding Remarks

We believe that our experience at the VCU Massey Cancer Center supports the use of HDR brachytherapy for cases of recurrent vulvar cancer. This technique is particularly useful for patients who have previously undergone surgery or radiation and/or for those that are not optimal surgical candidates. The patients discussed here had very unique cases that involved multiple recurrences and/or extensive disease both of which are extremely difficult to treat. This required a creative approach including atypical implant devices and a modified template. Ultimately, our experience could be adapted to other patients with difficult recurrent vulvar cancer and, in some cases, may serve as a guide to other providers. Because of our implementation of interstitial brachytherapy, we were able to increase and prolong patient comfort, and our approach ultimately allowed for preservation of a rectum in one patient preventing the need for colostomy. This technique is both safe and effective allowing for adequate disease control with improved quality of life for patients.

## Author Contributions

CM and EF were responsible for study conception and design and acquisition of data. KH, CM, and EF were responsible for the analysis and interpretation of data, drafting of manuscript, and revision.

## Conflict of Interest Statement

The authors declare that the research was conducted in the absence of any commercial or financial relationships that could be construed as a potential conflict of interest.

## References

[1] NCCN Guidelines. (2017). Available from: https://www.nccn.org/professionals/physician_gls/pdf/vulvar_blocks.pdf

[2] National Cancer Institute. (2017). Available from: https://www.cancer.gov/types/vulvar/hp

[3] Heaps JM Fu YS Montz FJ Hacker NF Berek JS. Surgical-pathologic variables predictive of local recurrence in squamous cell carcinoma of the vulva. Gynecol Oncol (1990) 38(3):309–14. 10.1016/0090-8258(90)90064-R 2227541

[4] Homesley HD Bundy BN Sedlis A Adcock L. Radiation therapy versus pelvic node resection for carcinoma of the vulva with positive groin nodes. Obstet Gynecol (1986) 68(6):733–40. 3785783

[5] Maggino T Landoni F Sartori E Zola P Gadducci A Alessi C Patterns of recurrence in patients with squamous cell carcinoma of the vulva. A multicenter CTF study. Cancer (2000) 89(1):116–22. 10.1002/1097-0142(20000701)89:1<116::AID-CNCR16>3.0.CO;2-4 10897008

[6] Mahner S Jueckstock J Hilpert F Neuser P Harter P de Gregorio N Adjuvant therapy in lymph node-positive vulvar cancer: the AGO-CaRE-1 study. J Natl Cancer Inst (2015) 107(3):dju426. 10.1093/jnci/dju426 25618900 PMC4356703

[7] Mahner S Prieske K Grimm D Trillsch F Prieske S von Amsberg G Systemic treatment of vulvar cancer. Expert Rev Anticancer Ther (2015) 15(6):629–37. 10.1586/14737140.2015.1037837 25997120

[8] Fonseca-Moutinho JA Recurrent vulvar cancer. Clin Obstet Gynecol (2005) 48(4):879–83. 10.1097/01.grf.0000179671.98939.fe 16286834

[9] Salom EM Penalver M. Recurrent vulvar cancer. Curr Treat Options Oncol (2002) 3(2):143–53. 10.1007/s11864-002-0060-x 12057077

[10] Kellas-Sleczka S Bialas B Fijalkowski M Wojcieszek P Szlag M Cholewka A Interstitial high-dose-rate brachytherapy in locally advanced and recurrent vulvar cancer. J Contemp Brachytherapy (2016) 8(1):32–40. 10.5114/jcb.2016.58081 26985195 PMC4793072

[11] Mahantshetty U Naga P Engineer R Sastri S Ghadi Y Upreti U Clinical outcome of high-dose-rate interstitial brachytherapy in vulvar cancer: a single institutional experience. Brachytherapy (2017) 16(1):153–60. 10.1016/j.brachy.2016.10.003 27876410

[12] Tewari K Cappuccini F Syed AM Puthawala A DiSaia PJ Berman ML Interstitial brachytherapy in the treatment of advanced and recurrent vulvar cancer. Am J Obstet Gynecol (1999) 181(1):91–8. 10.1016/S0002-9378(99)70441-2 10411801

[13] Viswanathan AN Szymonifka J Tempany-Afdhal CM O’Farrell DA Cormack RA. A prospective trial of real-time magnetic resonance-guided catheter placement in interstitial gynecologic brachytherapy. Brachytherapy (2013) 12(3):240–7. 10.1016/j.brachy.2012.08.006 23415048

[14] Hopkins MP Morley GW Pelvic exenteration for the treatment of vulvar cancer. Cancer (1992) 70(12):2835–8. 10.1002/1097-0142(19921215)70:12<2835::AID-CNCR2820701219>3.0.CO;2-U 1451064

[15] Angioli R Estape R Cantuaria G Mirhashemi R Williams H Martin J Urinary complications of Miami pouch: trend of conservative management. Am J Obstet Gynecol (1998) 179(2):343–8. 10.1016/S0002-9378(98)70362-X 9731836

[16] Dyk PT Richardson S Badiyan SN Schwarz JK Esthappan J Garcia-Ramirez JL Outpatient-based high-dose-rate interstitial brachytherapy for gynecologic malignancies. Brachytherapy (2015) 14(2):231–7. 10.1016/j.brachy.2014.11.017 25556865

[17] Pohar S Hoffstetter S Peiffert D Luporsi E Pernot M. Effectiveness of brachytherapy in treating carcinoma of the vulva. Int J Radiat Oncol Biol Phys (1995) 32(5):1455–60. 10.1016/0360-3016(95)00109-C 7635788

[18] Hoffman M Greenberg S Greenberg H Fiorica JV Roberts WS LaPolla JP Interstitial radiotherapy for the treatment of advanced or recurrent vulvar and distal vaginal malignancy. Am J Obstet Gynecol (1990) 162(5):1278–82. 10.1016/0002-9378(90)90036-7 2339729

[19] Prempree T Amornmarn R. Radiation treatment of recurrent carcinoma of the vulva. Cancer (1984) 54(9):1943–9. 10.1002/1097-0142(19841101)54:9<1943::AID-CNCR2820540926>3.0.CO;2-Z 6478428

[20] Castelnau-Marchand P Escande A Mazeron R Bentivegna E Cavalcanti A Gouy S Brachytherapy as part of the conservative treatment for primary and recurrent vulvar carcinoma. Brachytherapy (2017) 16(3):518–25. 10.1016/j.brachy.2017.01.005 28262516

[21] Podratz KC Symmonds RE Taylor WF. Carcinoma of the vulva: analysis of treatment failures. Am J Obstet Gynecol (1982) 143(3):340–51. 10.1016/0002-9378(82)90823-7 7081350

[22] Boronow RC. Combined therapy as an alternative to exenteration for locally advanced vulvo-vaginal cancer: rationale and results. Cancer (1982) 49(6):1085–91. 10.1002/1097-0142(19820315)49:6<1085::AID-CNCR2820490605>3.0.CO;2-4 7059935

[23] Boronow RC Hickman BT Reagan MT Smith RA Steadham RE. Combined therapy as an alternative to exenteration for locally advanced vulvovaginal cancer. II. Results, complications, and dosimetric and surgical considerations. Am J Clin Oncol (1987) 10(2):171–81. 10.1097/00000421-198704000-00055 3565317

